# Hemodialysis Experience After Kahramanmaraş Earthquake

**DOI:** 10.3390/jcm13216610

**Published:** 2024-11-04

**Authors:** Bulent Kaya, Mustafa Balal, Neslihan Seyrek, Burak Mete, Ibrahim Karayaylali

**Affiliations:** 1 Department of Nephrology, Faculty of Medicine, Çukurova University, Adana 01330, Turkey; mustafa.balal@gmail.com (M.B.); nseyrek12@gmail.com (N.S.); ibrahimk42@gmail.com (I.K.); 2 Department of Health Public, Faculty of Medicine, Çukurova University, Adana 01330, Turkey; burakmete2008@gmail.com

**Keywords:** crush syndrome, acute kidney injury, hemodialysis

## Abstract

**Background**: Hemodialysis treatment for acute kidney injury associated with crush syndrome is very complex. In our study, we summarized the problems and complications experienced by our hemodialysis center after the Kahramanmaraş earthquake. **Methods**: After the earthquake, our hospital treated 1396 victims. We evaluated the initial indications for dialysis, hemodialysis complications and the mortality of patients undergoing hemodialysis, including crush-related acute kidney injury (*n* = 82), during the earthquake period. We also compared them with patients who were undergoing hemodialysis (*n* = 76) in the same period but had end-stage renal failure and acute kidney injury due to other causes (*n* = 15). **Results**: After the earthquake, 173 adult patients, 91 (52.6%) of whom were male, with a mean age of 49.5 + 19.7 years, underwent hemodialysis between 6 and 22 February 2023. Patients with crush-related acute kidney injury experienced more complications during hemodialysis, and the increase in creatine kinase activity increased the risk of hemodialysis complications. The most common complications were blood clots in the dialyzer membrane, intradialytic hypotension, and intradialytic insufficient flow. The most frequent indication for initial hemodialysis was hyperkalemia (61, 74.4%). The major problems in the hemodialysis center included inadequate equipment and an insufficient number of experienced health personnel. **Conclusions**: Hyperkalemia is the most important initial indication for hemodialysis in patients with crush-related acute kidney injury. Crush-related acute kidney injury patients require hemodialysis more frequently, and hemodialysis complications are higher in patients with crush-related AKI, so the hemodialysis treatment of these patients should be more cautious. In an earthquake, hemodialysis centers may face significant challenges, such as damage, transportation issues, power outages, and water outages, which can hinder hemodialysis treatment.

## 1. Introduction

**Figure 1:**
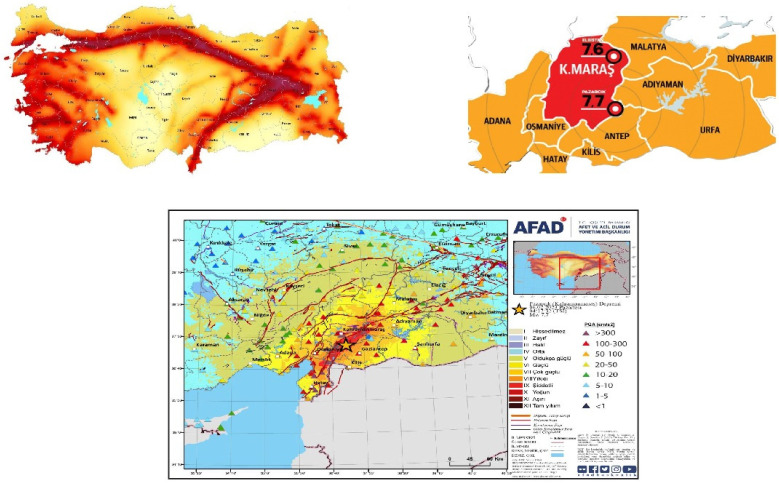
The earthquake danger map in Turkey displays the locations of earthquakes [4].

On 6 February 2023, an earthquake with a magnitude of 7.8 occurred in the Pazarcık province of Kahramanmaraş, in southern Turkey, near the northern border with Syria. About 9 h after this earthquake, at 13:24, another 7.5-magnitude earthquake followed in Elbistan province, about 59 miles (95 km) to the southwest. The two major earthquakes damaged an area of about 350,000 km^2^ (140,000 square miles) and affected 14 million people. In 11 city provinces in Turkey, a total of 518 thousand houses suffered severe or complete destruction (Figure 1). Additionally, 128 thousand 778 homes were moderately damaged. The disaster has displaced more than 2 million people, and at least 5 million have migrated to different provinces. The earthquakes resulted in at least 53,537 deaths in Turkey and at least 8476 deaths in Syria, along with more than 122,000 injuries. The first earthquake in Pazarcık was recorded as the largest earthquake in the Republic of Turkey’s history. The second earthquake in Elbistan was the third-largest earthquake among the earthquakes in Turkey. It is the deadliest earthquake since the 2010 Haiti earthquake, which killed over 300,000 people [1,2,3].

Severe trauma and compression following an earthquake cause death, while crush syndrome affects survivors [5,6]. Crush syndrome is a systemic manifestation of traumatic muscle injury. Direct trauma or ischemia damage the myocyte wall, releasing cell parts like myoglobin, potassium, phosphorus, and uric acid into the bloodstream after reperfusion. Also, after a crush injury, a lot of fluid can stay in the damaged muscles. This can cause compartment syndrome, low blood pressure, and poor blood flow to the kidneys, which can lead to acute kidney injury (AKI). Quickly administering the appropriate amount of fluid can prevent this [7,8]. As a result, a number of fatal and non-fatal complications can occur, including AKI, hypotension, acidemia, arrhythmia, acute respiratory distress syndrome, shock, and disseminated intravascular coagulation [5,8].

The inaccessibility of hospitals and hemodialysis (HD) centers for patients with earthquake-related crush injuries in need of acute HD treatment and end-stage renal disease (ESRD) for routine HD treatment are critical problems.

Many challenges can disrupt HD treatment, including damage to HD centers from earthquakes, issues with electricity and water, roadblocks, and a lack of transportation options. Additionally, a large number of earthquake victims apply to HD centers in the nearest region, causing a shortage of medical personnel, HD machines, pure water systems, and equipment [3,9].

In addition, the large number of earthquake victims in the closest HD centers causes shortages of medical personnel, HD machines, pure water systems, and equipment [5,10].

During the 1999 Marmara earthquake, 491 of 704 patients with AKI due to crush syndrome, most of whom were admitted to nearby centers, required HD [11].

Following the 2009 earthquake that damaged the town of L’Aquila in Italy and the surrounding area, 88 patients undergoing regular dialysis had to be transferred to the closest centers in order to continue undergoing HD treatment. Temporary dialysis centers were established in tents within three days to resolve these difficulties [9].

The Çukurova University Faculty of Medicine Hospital, a 1200-bed hospital located in Adana, 100 km from the earthquake-affected cities of Kahramanmaras, has a HD center with 18 HD machines, 4 doctors, 12 nurses, and 7 personnel. Çukurova University HD Center provides routine HD treatment for ESRD patients, as well as emergency HD for hospitalized and emergency department patients. Following the 6 February 2023 Kahramanmaraş earthquake, our hospital received and treated numerous earthquake victims. In addition to hospitalized patients with AKI and routine HD patients with ESRD, our center provided HD treatment to a very high number of patients in the short term, including earthquake victims with crush-related AKI. In this study, we summarized the problems and complications we experienced after referring to a large number of patients with crush syndrome requiring HD to our HD center.

## 2. Materials and Methods

**Figure 2:**
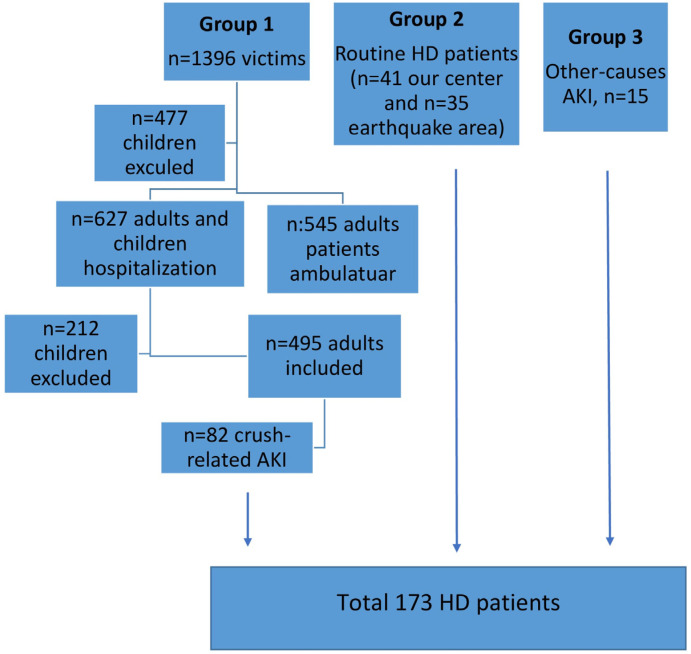
A flow chart of the study participants is shown.

We present a hospital record-based retrospective study among earthquake victims admitted to our hospital between 6 and 22 February 2023. After the Kahramanmaraş earthquake, our hospital treated 1.396 earthquake victims, the majority of whom were from Hatay and Kahramanmaras in Turkey. Among these patients, we identified patients who underwent HD due to crush-related AKI (82 patients) and patients who underwent HD due to ESRD (35 patients). We also identified patients with regular follow-up at our center who underwent HD due to ESRD (41 patients) and transient HD due to AKI due to other causes (unrelated to crush syndrome, 15 patients). Our study excluded patients under the age of 18 and those receiving continuous renal replacement therapy in intensive care units. Figure 2 displays the flow chart of the study participants.

All clinical, laboratory, and HD-related documents for patients were obtained from our hospital’s patient file system. We recorded the results of laboratory tests in the initial presentation, including glucose, blood urea nitrogen, creatinine, uric acid, AST, ALT, albumin, LDH, sodium, potassium, calcium, phosphorus, creatinine kinase, myoglobin, C-reactive protein, procalcitonin, whole blood count, and blood gas.

In patients with crush-related AKI, we identified indications for the first HD. The indications for HD in patients with AKI are volume overload, electrolyte imbalance (especially hyperkalemia), uremic symptoms, and metabolic acidosis [12]. For patients with crush syndrome, we classified the initial HD indications as follows: (1) hyperkalemia (serum K level > 6 meq/L) and crush-related AKI (serum creatinin > 3× baseline) and/or oliguria and/or metabolic acidosis; and (2) hypervolemia and crush-related AKI (serum creatinin > 3× baseline) and/or oliguria and/or metabolic acidosis.

The first HD treatment was performed for 2 h. We preferred HD solutions to contain potassium (K) 2 mmol/L, calcium (Ca) 1.5 mmol/L, HCO3 32 mmol/L and dextrose 1 g/L for initial HD.

If applicable, the femoral vein was the first HD access site in all crush-related AKI cases. All patients used similar types of HD devices (Fresenius 4008S HD Machine, Flowery Branch, GA, USA), HD catheters (NovaHF Hollow Fiber Dialyzer, Baxter Healthcare, Deerfield, IL, USA), and sets (Novaline, Tubing Sets for HD, Vital Healthcare, Wernersville, PA, USA). We recorded the frequency and duration of HD sessions. During all HD sessions, we recorded complications.

The study was approved by the Çukurova University Faculty of Medicine (04/2024, 143). All methods were performed in accordance with the Declaration of Helsinki. Patients were informed about this study and written informed consent was obtained.

We analyzed the data using the SPSS 20 (IBM, Armonk, NY, USA) software. We presented quantitative data as mean and standard deviation, and categorical data as number percentages. We used the Kolmogorov–Smirnov test as a normal distribution test. We analyzed the data using the *t*-test, chi-square test, and binary logistic regression analysis. We calculated the CK value exponentially among the variables included in the logistic regression analysis because there were numerous outliers. A *p*-value of <0.05 was considered statistically significant.

## 3. Results

We included 173 adult patients with a mean age of 49.5 + 19.7 years, including 91 (52.6%) males, who had undergone at least one HD treatment within a 15-day period from the first day of the earthquake. We divided these patients into three groups: crush-related AKI (*n* = 82, group 1), crush-unrelated AKI (from other causes, *n* = 15, group 2), and ESRD (*n* = 76, group 3). Table 1 displays the demographic information of the patients.

All patients underwent 509 HD treatments during this time, involving approximately 1628.1 h. The HD duration and distribution by group were as follows: ESRD (159 sessions, 532 h), crush-unrelated AKI (27 sessions, 79 h), and crush-related AKI (323 HD sessions, 1017.1 h).

Table 1 displays the distribution of patients according to the clinic where they started dialysis. A significant proportion of patients with crush-related AKI (44, 53.7%) were hospitalized in the nephrology clinic.

In the post-earthquake period, between 6 and 22 February 2023, 6 out of the 173 patients (five crush-related AKI and other-cause AKI) who underwent HD died (Table 1).

Table 1 also categorizes the types and numbers of complications during HD, according to the groups. The most common ones were blood clots in the dialyzer membrane (35 patients, or 20.2%), intradialytic hypotension (17 patients, or 9.8%), intradialytic insufficient flow (15 patients, or 8.7%), and fever (8 patients, or 4.6%).

**Figure 3:**
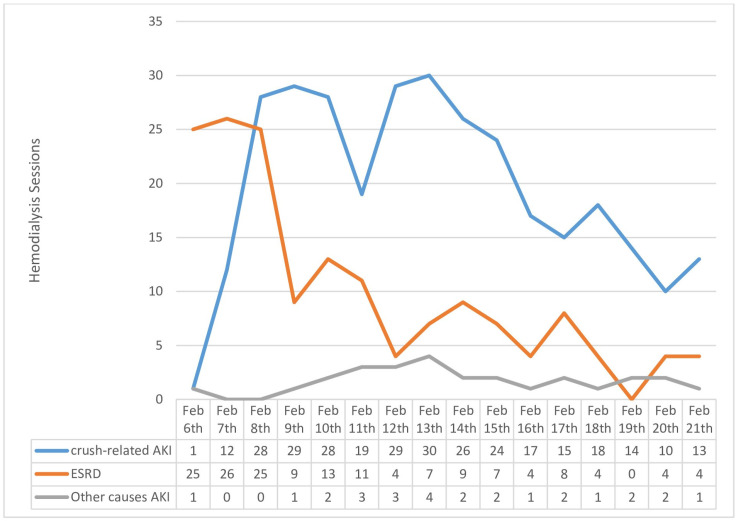
Number of daily hemodialysis sessions between 6 and 21 February 2023 (*n* = 173).

Figure 3 shows the daily number of patients who underwent HD treatment between 6 and 21 February 2023. The second day following the earthquake had the highest number of HD treatments, with a total of fifty-three HD (28 crush-related AKI, 25 ESRD) treatments.

Table 2 shows the total number of complications observed during HD across all sessions. Patients with crush-related AKI experienced a higher number of complications during HD.

There were two main groups of people with initial HD indications in crush-related AKI: those with hyperkalemia (61, 74.4%) and those with hypervolemia (21, 25.6%). Table 3 shows the distribution of initial indications for HD in patients with crush-related AKI according to causes and clinics. The initial indications for HD in patients who underwent crush-related AKI did not differ significantly between the three groups (nephrology service, intensive care unit, and emergency department). Additionally, the clinics (nephrology service, intensive care unit, and emergency department) found no significant difference in HD complications among patients who underwent HD for crush-related AKI.

Table 4 presents a comparison of HD indications based on the mortality status of patients with crush-related AKI. There was no significant difference between patients who died from crush-related AKI according to the initial indication.

Table 5 shows the initial laboratory values of patients undergoing HD for crush-related AKI. We included biochemical parameters as independent variables in the regression analysis to estimate the risk of complications in patients undergoing HD due to crush-related AKI (Table 6). The model was significant (omnibus test *p* < 0.001) and had an accuracy rate of 69.6%, sensitivity of 65.8%, and specificity of 73.2%. Our findings showed that the complication risk increased 5.1-fold for every 110-unit increase in the creatine kinase enzyme (log CK).

Table 7 shows the locations of trauma, and the surgical procedures performed in patients undergoing HD for crush-related AKI. As a result, the most common trauma site was the lower extremities, and the most common surgical procedure was limb amputation.

## 4. Discussion

Following the Kahramanmaraş earthquake, we transformed a medical faculty hospital into a trauma hospital. Our hospital received 1396 patients (919 adults) between 6 and 21 February 2023; hospitalized 627 (415 adults) of them; and provided HD treatment for 82 (8.9% of all adult patients and 19.7% of hospitalized adult patients) of them at least once due to crush-related AKI.

Following the earthquake, the main problems in our HD Center were the large number of patients requiring HD, as well as an inadequate number of HD devices, equipment, pure water systems and medical personnel. Our hospital, which has a capacity of 1200 patients, has an HD center with 18 HD devices. This center typically provides HD services to 41 ESRD patients three times a week for four hours in three shifts, as well as to patients with AKI hospitalized in clinics or emergency departments through night call shifts. In addition to our routine HD patients, this number has reached 76, with 35 ESRD patients coming from HD centers in the earthquake region as of 6–21 February 2023. One of the important problems is that patients receiving routine dialysis treatment cannot reach a suitable HD center after the earthquake. The primary issues following the earthquake included a lack of water, electricity, transportation, and the destruction of HD centers [10]. Since our hospital and HD center were close to the earthquake zone, patients were able to reach our center. Following the 2009 earthquake in L’Aquila, Italy, which left 308 people dead and nearly 1500 injured, the town’s HD center suffered significant damage. As a result, 88 routine HD patients there had difficulties receiving HD treatment, necessitating their transfer to nearby HD centers. Fortunately, they were able to overcome these problems by setting up a temporary HD center in a tent with 13 HD machines in a short time [9]. In 2011, the Great Tohoku earthquake and subsequent tsunami caused severe devastation in Japan. Patients with ESRD were unable to receive dialysis in the disaster zone due to drug shortages, water, and power outages. Hospitals in the neighboring earthquake-affected region were unable to accept these patients due to the shortage of medical resources. Seventeen doctors also suffered from the disaster, caring for ESRD patients. Despite the challenging conditions, they successfully transferred 584 dialysis patients to centers and hospitals in the region without any patient deaths [3]. Similarly, more than 2400 dialysis patients experienced severe difficulties after the collapse of many chronic dialysis units [13].

It is difficult to estimate the number and severity of injured people who may re-quire AKI and HD after an earthquake. The structure of the buildings in the area, the speed of rescue, the administration of intravenous fluids to the injured at the location, and the intensity and timing of the earthquake can affect the number of crush-related AKI victims. In 1988, the Armenia earthquake caused around 150,000 deaths. After arriving at the disaster site with a significant delay, rescuers rescued around 600 injured people from under the rubble, many of whom developed AKI and died due to a lack of HD [14]. In the 1999 Marmara earthquake, 477 of the 639 registered crush syndrome patients required dialysis treatment, and >5000 dialysis treatments were performed [15,16].

The number of earthquake victims in need of dialysis who were transported to our hospital after the earthquake significantly exceeded the capacity of our HD center. Due to the shortage of disposable dialysis materials (dialyzers, needles, serum sets, and concentrated solutions), we asked for help from other hospitals and the Turkish Nephrology Association. There was no mobile water purification system in our hospital, and the number of HD machines was insufficient, so we requested urgent help from international relief organizations and the Turkish Nephrology Association. Although the earthquake affected a huge area, we were able to receive most of our urgent requests for help.

Our HD center was treating routine HD patients in three shifts and providing HD treatment to AKI patients as needed. However, due to an excessive number of patients during the earthquake period, the HD center provided a continuous HD service throughout the day. Furthermore, physicians, nurses, and supportive medical personnel with HD experience are required to manage hemodialysis treatment effectively. The transfer of experienced health personnel with HD experience from other clinics and the contribution of volunteer health personnel are essential to the effective treatment of HD patients, including earthquake victims [9,10]. By transferring nurses, doctors, and supportive health personnel from other clinics within our hospital, we resolved this issue. In addition, with the contribution of volunteer health personnel working in other institutions in Turkey, earthquake victims were able to undergo appropriate HD treatment. Our hospital and HD center’s proximity to Kahramanmaraş, the epicenter of the earthquake, made it possible for earthquake victim patients to reach us. However, as a result of technical research between 6 and 22 February 2023, our hospital was closed due to mid-level damage and then patients were transferred to other health centers in the city.

During this period, we treated 97 adult AKI patients (82 with crush related AKI and 15 with other causes of AKI), hospitalized them in the nephrology service and intensive care units, and provided HD treatment at least once. About one-third (74.4%) of crush-related AKI patients required dialysis due to hyperkalemia, and 25.6% required hypervolemia.

HD is lifesaving for patients with AKI. Although the early initiation of HD seems intuitively beneficial, the evidence base is inconsistent. It is common for the biochemical signs of solute imbalance (azotemia, hyperkalemia, and severe metabolic acidosis) and the clinical signs of volume overload to be checked before starting renal replacement therapy [7]. Data on HD frequency and efficacy are also controversial; frequent HD may or may not improve the final outcome [17]. AKI from crush syndrome, on the other hand, is more often linked to fluid overload, hypercatabolism, acidosis, uremia, and life-threatening hyperkalemia than AKI from other causes. Therefore, given the complications, there are fewer restrictions on starting HD in crush syndrome patients compared with patients with other causes of AKI. Not only hyperkalemia but also rapidly increasing serum potassium may be an indication of HD [7,18]. We performed HD on our patients with crush syndrome, closely monitoring the severity of trauma, renal function and electrolytes, daily urine volumes, and clinical clues. We did not perform prophylactic HD, but we can say that these patients require HD more frequently than patients with AKI due to other causes.

Our patients with routine ESRD underwent HD treatment 3 days a week for 3 h instead of 3 days a week for 4 h. Due to the large number of patients, some even underwent HD for two hours. We informed the patients to adhere to fluid and dietary restrictions, and during the 15-day period, no patient needed additional HD treatment for urgent indications like hypervolemia or hyperkalemia. Reports during the earthquake suggested that strict adherence to dietary and fluid restrictions could reduce the number of HD sessions [10]. Following the Great Tohoku Earthquake, 584 dialysis patients waited a median of 3 days between dialysis sessions during transport and searched for a nearby center. Although patients experienced hyperkalemia and volume overload, there were no deaths [3]. This result shows that in extraordinary situations such as disasters, we can treat routine HD patients with HD for a shorter period of time with effective patient education. We should educate ESRD patients in earthquake-affected areas on how to behave during a disaster, particularly if their treatment unit sustains damage.

Hyperkalemia is one of the issues that requires particular caution in patients who develop AKI due to crush syndrome [19,20]. Damage to the muscle cell membrane causes an excessive rise in potassium levels in the blood because cell contents such as potassium, uric acid, phosphorus, and lactate are released [21]. Concomitant metabolic acidosis, hyperphosphatemia, and hypocalcemia can exacerbate fatal arrhythmias caused by hyperkalemia [8,22]. Hyperkalemia was an indication in a significant proportion (74.4%) of patients undergoing HD for the first time, particularly in the emergency department (88.2%). To prevent arrhythmias, we used a serum potassium level of dialysate of 2 mmol/L instead of 1 mmol/L, and to avoid hypocalcemia, we used a dialysate calcium level of 1.5 or 1.75 mmol/L instead of 1.25 mmol/L. We preferred bicarbonate HD solutions (32 mmol/L) to correct acidosis, as we rarely use acetate in dialysate.

Because of the significant number of patients, especially in the early period of the earthquake, we adjusted the duration of HD to 2 h and the potassium concentration in the HD solution to 2 mmol/L. Low dialysate potassium levels can cause arrhythmias [23]. We were concerned about whether both short-term HD and adjusting the serum potassium level to 2 mmol/L would sufficiently reduce the serum potassium level. We checked patients’ serum potassium levels at least twice a day, and no patient required re-HD due to hyperkalemia on the same day.

In earthquake victims rescued with a delay of several days, a more restricted fluid therapy approach is required, taking into account the possibility of anuria. In the Marmara earthquake, more fluids were infused into victims in need of HD because these victims were admitted several days after the disaster without a urinary response to fluids, resulting in hypervolemia and a high need for HD [7]. Hypervolemia, which was present in 25.6% of our patients with crush-related AKI, was another indication for HD. There was no difference in the indications for hypervolemia in the emergency, nephrology service, or critical care units. The late rescue of patients from the earthquake, late access to our hospital, or uncontrolled fluid treatments may result in hypervolemia. Hypervolemia is one of the factors that negatively affects renal and all-cause survival [24]. Patients in the critical care unit, where fluid therapy is more closely monitored, were expected to undergo HD less frequently with the indication of hypervolemia, but we found no difference between clinics. We assessed the patients’ volume status by looking at their daily fluid intake and output, their weight (if possible), and symptoms such as shortness of breath. In addition, we performed a physical examination, looking for pretibial edema, fine rales in the lungs, neck vein distension, and other conditions. In the chest x-rays, we evaluated the cardiothoracic ratio, cardiothoracic sinuses, and Kerley B lines. Time limitations, a lack of experienced personnel, and a lack of appropriate devices and infrastructure prevented us from performing techniques such as echocardiography, inferior vena cava diameter, body fluid monitoring with a Body Composition Monitor (BCM), and lung ultrasonography, which can determine volume status [25,26,27]. Fluid therapy should receive special concern in these patients because hypervolemia is the first indication for HD in about 25.6% of patients with crush-related AKI, especially in the critical care unit and nephrology service.

A previous study, like ours, found that the most common complication in patients with crush-related AKI was clots in the dialysis membrane during HD [28]. This may be caused by planned surgical procedures resulting from amputation and compartment syndrome, non-heparinized dialysis due to bleeding from fasciotomy and debridement, or hypotension. The second most common complication was inadequate blood flow for dialysis. This may be due to problems with catheter placement, dialysis without heparin, membrane clotting, and hypotension [28].

Hypotension from excessive fluid removal during HD and electrolyte abnormalities typically induce muscle cramps [29]. Our patients had a low incidence of cramps, likely due to their effective intravenous rehydration, the short duration of HD, and the weak cramping mechanism of painfully damaged muscles.

Only one crush-related AKI patient experienced cardiac arrest during HD, necessitating urgent resuscitation, intubation, and transport to the intensive care unit. The number of complications in crush-related AKI patients across all HD sessions did not differ between patients in the nephrology clinic, emergency department, or intensive care unit. However, we can say that crush-related AKI patients have a higher frequency of HD complications compared to routine HD patients, and closer follow-up and preventive applications are required. We also discovered that the risk of complications during HD increases with elevated levels of creatine kinase enzyme, an indicator of muscle damage. We can say that less muscle damage is associated with fewer HD complications, and taking measures to reduce the risk of muscle damage (surgical or medical, such as fasciotomy in compartment syndrome or amputation when necessary) is critical.

Patients who develop AKI due to crush syndrome usually recover from it if the problems causing kidney failure are corrected [30,31]. However, complications such as hyperkalemia, profound metabolic acidosis, and shock can cause death in these patients [32,33,34]. Patients with crush-related AKI requiring dialysis have had mortality rates of 15–20% in various earthquakes [16,35,36,37].Five of the 86 patients with crush-related acute kidney injuries who received HD died. There is almost no risk of ESRD development in patients who develop crush-related AKI with appropriate medical or surgical treatments [30]. Crush-related AKI did not require long-term kidney replacement treatment in any of the patient’s undergoing hemodialysis.

Patients undergoing HD for crush-related AKI had the most commonly traumatized lower extremities (69.5%, 57). This result is similar to the Marmara earthquake [11]. The most common surgical procedures were amputation (30.5%, 25), fasciotomy (23.2%, 19), and debridement (9.8%, 8). The amputation rate was higher than in the Marmara earthquake [11]. The earthquake’s intensity, the large number of affected patients, the prolonged time under the collapsed building, and the transportation time could all contribute to this.

This study has several limitations. For instance, the closure of our hospital due to damage necessitated our relocation to a different facility, thereby complicating data access. The closure of the hospital caused difficulties in following up with existing patients. Due to the numerous patients applying at the same time, we were unable to provide accurate laboratory and imaging methods.

## 5. Conclusions

Measures to reduce the risk of muscle damage, such as providing early rescue for earthquake victims, accelerating hospital transport, and applying early surgical interventions, are effective in reducing crush-related AKI, HD needs, and HD complications.

Hyperkalemia is the most important initial indication for hemodialysis in patients with crush-related acute kidney injury. Crush-related acute kidney injury patients require hemodialysis more frequently, and HD complications are higher in patients with crush-associated AKI, so the HD treatment of these patients should be more cautious.

In disasters such as earthquakes, when the HD center is insufficient due to numerous dialysis patients, providing training or advice to routine HD patients about fluids and dietary restrictions can be a temporary solution for the center to ensure that services are provided to all patients.

Due to the significant number of crush-related AKI patients admitted to hospitals after an earthquake disaster, there may be shortages of medical equipment, devices, and medical personnel to provide services to these patients. Dialysis patients may also face significant complications that hinder HD treatment, such as the destruction of HD centers, transportation issues, power outages, and water shortages. In this situation, we must design HD centers with an expandable capacity, resilience to seismic events, and readiness for challenges related to infrastructure and medical staff. It is important that local, national, and international help and support are available in disaster situations to ensure appropriate treatment. To ensure rapid and appropriate treatment in these scenarios, we must prepare in advance by creating an emergency action plan and design its elements under the umbrella of disaster nephrology. To prepare for disaster scenarios, we should design HD centers with expandable capacity, resistance to seismic events, and sufficient medical equipment, HD devices, and health personnel.

## Figures and Tables

**Table 1:** Demographic characteristics of patients undergoing hemodialysis (*n* = 173).

Parameters	Crush-Related AKI (*n* = 82) *n* (%) or Mean ± SD (Min–Max)	End-Stage Renal Disease (*n* = 76) *n* (%) or MEAN ± SD (Min–Max)	AKI from Other Causes (*n* = 15) *n* (%) or Mean ± SD (Min–Max)	*p*-Value
Sex				
Male Female	45 (54.9) 37 (45.1)	37 (48.7) 39 (51.3)	9 (60) 6 (40)	0.616
Years	37.9 ± 14.6 (19–75)	58.2 ± 18.3 (21–87)	68.7 ± 13.1 (51–94)	<0.01
Total number of HD sessions	323	159	27	
Total number of HD sessions	3.94 ± 3.3 (1–11)	2.09 ± 1.7 (1–7)	1.8 ± 1.7 (1–7)	<0.01
Total session duration of HD, hours	1017.1	532	79	
Total session duration of HD, hours	12.4 ± 11.2 (1–40)	7 ± 5.5 (1.5–40)	5.2 ± 6.7 (1–25)	<0.001
Survival				
Exitus Alive	5 (6.1) 77 (93.9)	- 76 (100)	1 (6.7) 14 (93.3)	0.087
Clinic				
Emergency clinic Nephrology clinic Critical care clinic Routine HD clinic	17 (20.7) 44 (53.7) 21 (25.6) -	0 1 (1.3) - 75 (98.7)	10 (66.7) 2 (13.3) 3 (20) -	<0.01
Catheter type				
Transient catheter Tunneled catheter Arteriovenous fistula	82 (100) - -	- 24 (31.6) 52 (68.4)	15 (100) - -	<0.01
HD Complications counts, y/n				
Intradialytic hypotension	9/323	2/159	6/27	
Intradialytic low flow count	13/323	-	2/79	
Clotting in the HD membrane	32/323	1/159	2/79	
Intradialytic fever	8/323	-	-	
Intradialytic leaks	1/323	-	-	
Intradialytic arrest	1/323	-	-	
Intradialytic hypertension	1/323	-	-	
Intradialytic cramp	-	1/159	-	
Intradialytic seizures	1/323	-	-	
Intradialytic nausea	1/323	-	-	

AKI: acute kidney injury, HD: hemodialysis, y/n: yes/no.

**Table 2:** Hemodialysis complications according to etiology in all patients (*n* = 173).

Parameters	Crush-Related AKI (*n* = 82) *n* (%)	End-Stage Renal Disease (*n* = 76) *n* (%)	AKI from Other Causes (*n* = 15) *n* (%)	*p*-Value
Number of complications				
0	41 (50)	73 (96.1)	6 (40)	<0.01
1	21 (25.6)	2 (2.6)	7 (46.7)	
2	17 (20.7)	1 (1.3)	2 (13.3)	
3	2 (2.4)	0	0	
4	1 (1.2)	0	0	
Number of intradialytic hypotension, y/n	9 (11)/73 (89)	2 (2.6)/74 (97.4)	6 (40)/9 (60)	<0.01
Intradialytic low flow count, y/n	13 (15.9)/69 (84.1)	0/76 (100)	2 (13.3)/13 (86.7)	<0.01
Clotting in the HD membrane, y/n	32 (39)/50 (61)	1 (1.3)/75 (98.7)	2 (13.3)/13 (86.7)	<0.01

AKI: acute kidney ınjury, HD: hemodialysis, y/n: yes/no.

**Table 3:** Number of indications and complications according to crush-related AKI patients in clinics (*n* = 82).

	Nephrology Clinic (*n* = 44), *n* (%)	Critical Care Clinic (*n* = 21), *n* (%)	Emergency Clinic (*n* = 17), *n* (%)	Overall (*n* = 82), *n* (%)	*p*-Value
Indication					
Hyperkalemia + X	31 (70.5)	15 (71.4)	15 (88.2)	61 (74.4)	0.339
Hypervolemia + X	13 (29.5)	6 (28.6)	2 (11.8)	21 (25.6)	
Number of HD complications					
0	24 (54.5)	9 (42.9)	8 (47.1)	44	0.245
1	13 (29.5)	3 (14.3)	5 (29.4)	21	
2	6 (13.6)	8 (38.1)	3 (17.6)	17	
3	1 (2.3)	0	1 (5.9)	2	
>4	0	1 (4.8)	0	1	

AKI: acute kidney injury, X: crush-related AKI (serum creatinine > 3× baseline) and/or oliguria and/or metabolic acidosis.

**Table 4:** Comparison of hemodialysis indication according to mortality status for crush-related AKI patients (*n* = 82).

	Survival *n* (%)			
Hemodialysis Indication	Exitus	Alive	Overall	*p*-Value
Hyperkalemia + X	4 (6.6)	57 (93.4)	61 (100)	1.000
Hypervolemia + X	1 (4.8)	20 (95.2)	21 (100)	

AKI: acute kidney injury, X: crush-related AKI (serum creatinine > 3× baseline) and/or oliguria and/or metabolic acidosis.

**Table 5:** Laboratory findings at initial presentation of crush-related AKI (*n* = 82).

Parameters	Mean + SD (Min–Max)
BUN, mg/dL	67.1 ± 38.2 (21.7–279)
Creatinine, mg/dL	3.44 ± 2.07 (0.76–14.93)
Maximum creatinine, mg/dL	5.2 ± 2.59 (0.76–14.93)
Uric acid, mg/dL	9.9 ± 3.9 (2.8–19.9)
AST, U/L	1012 ± 1262 (9–7429)
ALT, U/L	456 ± 769 (8–4519)
Albumin, g/L	27.3 ± 5.8 (12.8–39.2)
LDH, U/L	1930 ± 2068 (15–12,850)
Sodium, mmol/L	136 ± 8.2 (116–158)
Potassium, mmol/L	5.8 ± 1.2 (3.1–9.8)
Calcium, mg/dL	7.2 ± 1.0 (5.2–9.4)
Phosphorus, mg/dL	6.2 ± 2.6 (2.3–15.2)
CK, U/L	46,992 ± 5037 (44–315,124)
Maximum CK, U/L	91,374 ± 113,252 (44–744,410)
Myoglobin, ng/mL	3651 ± 1053 (57–4100)
Hgb, g/dL	13.5 ± 2.9 (7.4–20)
WBC, 10^3^/μL	19.8 ± 9.7 (5.7–43.7)
Plt, 10^3^/μL	217 ± 86 (69–513)
CRP, mg/L	159 ± 91 (29.6–475)
Procalcitonin, ng/mL	23.1 ± 29.4 (0.1–98)

AKI: acute kidney njury, CK: creatine kinase, CRP: C-reactive protein, Hgb: hemoglobin, LDH: lactate dehydrogenase, Plt: platelet, WBC: white blood count, AST: aspartate aminotransferase, ALT: alanine aminotransferase.

**Table 6:** Logistic regression analysis of complications during hemodialysis for patients with crush-related AKI (*n* = 82).

	B	Sig.	Exp(B)	95% C.I. for EXP(B)	
				Lower	Upper
BUN, mg/dL	−0.008	0.351	0.992	0.975	1.009
LDH, U/L	0.000	0.806	1.000	1.000	1.000
AST, U/L	0.000	0.668	1.000	0.999	1.000
Phosphorus, mg/dL	0.088	0.466	1.092	0.862	1.383
Potassium, mmol/L	0.400	0.112	1.491	0.911	2.440
Myoglobin, ng/mL	0.000	0.750	1.000	0.999	1.001
Procalcitonin, ng/mL	−0.006	0.530	0.994	0.974	1.014
CRP, mg/L	−0.001	0.746	0.999	0.993	1.005
Creatinine, mg/dL	0.187	0.314	1.206	0.837	1.736
Log CK	1.631	0.036	5.107	1.114	23.421
Albumin, g/L	0.083	0.097	1.086	0.985	1.198
Uric acid, mg/dL	−0.004	0.965	0.996	0.841	1.181
Constant	−12.183	0.002	0.000		

AKI: acute kidney injury, Log CK: logarithmic creatine kinase, CRP: C-reactive protein.

**Table 7:** Demographic data of patients undergoing hemodialysis treatment for crush-related AKI (*n* = 82).

Parameters	Values
Time trapped (h)	20 (0–138)
İnjury sites, *n* (%)	
Upper extremity	3 (3.7)
Lower extremity	57 (69.5)
Upper and Lower extremity	22 (26.8)
Pelvic trauma	3 (3.7)
Abdominal trauma	8 (9.8)
Chest trauma	12 (14.6)
Head trauma	2 (2.4)
Vertebral colon trauma	6 (7.3)
Number of fasciotomy with patients, *n* (%)	19 (23.2)
Number of debridman with patients, *n* (%)	8 (9.8)
Number of amputation with patients, *n* (%)	25 (30.5)

AKI: acute kidney injury.

## Data Availability

Data can be provided upon request.
